# Safety and early organ preservation outcomes after dose escalation with MR-guided radiotherapy for rectal cancer (preRADAR): A phase I trial

**DOI:** 10.1016/j.ctro.2026.101191

**Published:** 2026-05-25

**Authors:** H. Eijkelenkamp, M.E. Verweij, G. Grimbergen, M. Tanaka, C.M. Kensen, T. Janssen, T. Vijlbrief, W.M.U. van Grevenstein, L.M.G. Moons, M. Koopman, M.M. Lacle, M.N.G.J.A. Braat, M. Maas, E.C.J. Consten, A. Pronk, A.B. Smits, J.T. Heikens, S.G. Elias, H.M. Verkooijen, G.J. Meijer, A. Couwenberg, M.P.W. Intven

**Affiliations:** a Department of Radiation-Oncology, University Medical Centre Utrecht, Utrecht University, Utrecht, the Netherlands; b Department of Radiation-Oncology, Netherlands Cancer Institute, Amsterdam, the Netherlands; c Department of Surgery, University Medical Centre Utrecht, Utrecht University, Utrecht, the Netherlands; d Department of Gastroenterology, University Medical Centre Utrecht, Utrecht University, Utrecht, the Netherlands; e Department of Medical Oncology, University Medical Centre Utrecht, Utrecht University, Utrecht, the Netherlands; f Department of Pathology, University Medical Centre Utrecht, Utrecht University, Utrecht, the Netherlands; g Department of Radiology, University Medical Centre Utrecht, Utrecht University, Utrecht, the Netherlands; h Department of Radiology, Netherlands Cancer Institute, Amsterdam, the Netherlands; i Department of Surgery, Meander Medical Centre, Amersfoort, the Netherlands; j Department of Surgery, University Medical Centre Groningen, Groningen, the Netherlands; k Department of Surgery, Diakonessenhuis Utrecht Zeist Doorn, Utrecht, the Netherlands; l Department of Surgery, Sint Antonius Hospital, Nieuwegein, the Netherlands; m Department of Surgery, Hospital Rivierenland, Tiel, the Netherlands; n Department of Epidemiology & Health Economics, Julius Center for Health Sciences and Primary Care, University Medical Center Utrecht, Utrecht University, Utrecht, the Netherlands; o Division of Imaging and Oncology, University Medical Center Utrecht, Utrecht University, Utrecht, the Netherlands

**Keywords:** MR-guided radiotherapy, Dose escalation after short-course radiotherapy, Rectal cancer, Organ preservation, Phase I on safety and feasibility

## Abstract

**Background and purpose:**

Organ preservation is a desirable goal for patients with rectal cancer. Magnetic resonance-guided radiotherapy (MRgRT) enables precise dose delivery and online adaptation. This phase I study (preRADAR) evaluated maximum tolerated dose (MTD) of short course radiotherapy (SCRT; 5x5 Gy) with a subsequent boost using MRgRT in intermediate-risk rectal cancer.

**Materials and methods:**

the trial had a 6 + 3 dose-escalation design. Patients staged T3c-d(MRF-)N0M0 or T1-3(MRF-)N1M0 were treated on a 1.5 Tesla MR-linac with short-course radiotherapy (SCRT; 5 × 5 Gy) followed by 2–4 daily 5 Gy GTV boosts according to the dose level. Safety was assessed by incidence of acute and perioperative dose-limiting toxicities (DLTs) within prespecified time windows and stopping boundaries. Secondary endpoints included technical feasibility (boost PTV V95% > 90%) and organ preservation at six months.

**Results:**

Out of 31 patients screened, sixteen were treated across three dose levels. All fractions were delivered as planned and all adaptive boost fractions achieved the predefined feasibility criterion (PTV V95% >90%). No acute DLTs occurred. Three perioperative DLTs were observed, including two anastomotic leaks requiring reoperation. The MTD was not identified; dose level 2 (SCRT + 4 × 5 Gy boosts) was the highest dose level completed within the DLT stopping boundaries, achieving a 26-week organ preservation rate of 56% (5/9).

**Conclusion:**

MRgRT-based enables dose escalation following SCRT in intermediate-risk rectal cancer. The highest evaluated dose level, 4 × 5 Gy GTV boosts after SCRT, was considered safe within the predefined DLT boundaries and feasible for further evaluation.

## Highlights

• MR-guided SCRT with subsequent dose escalation was assessed for rectal cancer.
• Up to 4 additional 5 Gy GTV boosts after SCRT was safe and technically acceptable.
• 26-weeks organ preservation rate incrementally increased to 56% at that dose level.

## Introduction

Total mesorectal excision (TME) remains the predominant curative treatment for rectal cancer. Yet, it is associated with considerable perioperative morbidity and long-term functional impairment, including the need for temporary or permanent colostomy and bowel, urinary, and sexual dysfunction [1], [2], [3], [4]. In carefully selected patients, organ-preserving strategies – most notably active surveillance (watch-and-wait) after a clinical complete response (cCR) to neoadjuvant therapy – can omit TME from the treatment strategy. Organ preservation reduces treatment burden while maintaining oncologic safety, organ function, and quality of life [5], [6], [7], [8], [9], [10], [11], [12], [13].

Short-course radiotherapy (SCRT) followed by delayed surgery is a guideline-endorsed treatment option for localized rectal cancer. The Stockholm III trial demonstrated that postponing surgery after SCRT reduces postoperative complications without compromising oncologic outcomes. Moreover, the delay allows for response assessment and offers the potential for organ preservation [4], [14], [15], [16]. The observed number of cCR after radiotherapy is dose dependent [17]. SCRT alone yields a low rate of cCR. Higher delivered doses are associated with greater tumor regression [18]. Dose escalation is likely to increase the proportion of patients eligible for organ-preserving approaches, [19] but it also carries a higher risk of toxicity and functional morbidity, and therefore requires highly precise radiotherapy delivery techniques.

Magnetic resonance-guided radiotherapy (MRgRT) enables high-precision dose delivery with daily MRI, motion monitoring, and online adaptive replanning to correct for anatomical changes. These abilities permit tighter margins and dose escalation to the gross tumor volume (GTV) while respecting organs-at-risk [20], [21], [22], [23]. Early clinical experience on 1.5 Tesla (T) MR-linacs have confirmed workflow feasibility in rectal cancer. Prospective cohorts suggest favorable early outcomes and quality of life after MR-guided SCRT, supporting further investigations of adaptive, dose-escalated strategies [24], [25].

Accordingly, this phase I trial was designed to determine the maximum tolerated dose (MTD), safety, and technical feasibility of dose-escalated online adaptive MRgRT in patients with intermediate-risk rectal cancer undergoing neoadjuvant SCRT. The results of this trial are intended to guide future trials investigating MRgRT-based dose escalation strategies for organ preservation in rectal cancer.

## Materials and methods

The Towards Response ADAptive Radiotherapy for organ preservation for intermediate-risk rectal cancer – preRADAR – was a phase I trial (R-IDEAL stage 2a) that evaluated the safety and feasibility of dose-escalated SCRT administered with online adaptive MRgRT in patients with intermediate-risk rectal cancer [26]. A 6 + 3 dose-escalation design was chosen to permit early detection of dose-limiting toxicity (DLT) with a stricter safety threshold compared to a more conventional 3 + 3 design. The trial protocol was published previously [20]. The trial received approval from the institutional ethics committee and was registered in the WHO International Clinical Trials Registry (NL8997).

### Eligibility criteria

Patients were eligible if they had biopsy-confirmed cT3c-d(MRF-)N0M0 or cT1-3(MRF-)N1M0 rectal adenocarcinoma located entirely below the sigmoid take-off (mid or distal rectum), were referred for neoadjuvant SCRT to the University Medical Center Utrecht and were interested in organ preservation. Exclusion criteria included planned induction systemic therapy, inflammatory bowel disease, extramesorectal pathological lymph nodes, extramural venous invasion grade 3 or 4, prior pelvic radiotherapy, MRI contraindications, orthopedic hip implants and pregnancy. Written informed consent was obtained from all participants.

### Dose levels

**Fig. 1:**
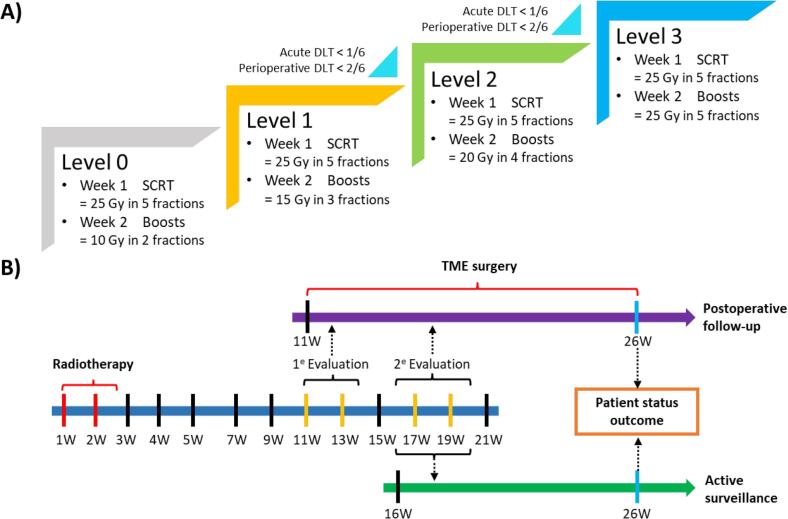
A) Study schema showing the four dose-levels, radiotherapy regimes and predefined escalation rules based on dose-limiting toxicity (DLT) incidence. B) Participant timeline with consultations, evaluation moments, and follow-up pathways. Blue line = acute DLT evaluation window; purple line = postoperative DLT evaluation window; green line = active surveillance period. (For interpretation of the references to colour in this figure legend, the reader is referred to the web version of this article.)

The trial consisted of four dose level with escalating boost dose. Patient enrollment started at dose level 1 (3 × 5 Gy boost). When a dose level was fully accrued but the DLT evaluation period was still ongoing, additional eligible patients could be enrolled at a lower dose level that had already been deemed safe (hence dose level 0; 2 × 5 Gy boost). The trial was originally designed with four dose levels, including a highest level with a 5 × 5 Gy boost. After a Dutch national guideline update removing SCRT as standard of care for intermediate-risk rectal cancer, accrual was no longer feasible. Consequently, the trial was closed prematurely after completion of dose level 2 (4 × 5 Gy boost). An illustration of the trial setup is shown in Fig. 1.

### Radiotherapy intervention

All patients underwent a planning MRI on a 3T scanner. In addition to the 3D T2-weighted sequence used for target delineation, radiotherapy planning used either an MR-derived pseudo-CT sequence from the same session or a separate CT scan. Radiotherapy was administered using a 1.5 T MR-linac (Elekta AB, Sweden). SCRT (5 × 5 Gy) was directed to the mesorectum and presacral and internal iliac lymph nodes regions using a planning target volume (PTV) margin of 6 mm ventrally and 4 mm in all other directions [24]. Additional boosts (2–4 × 5 Gy) targeted the gross tumor volume (GTV) and suspected lymph nodes with 5 mm isotropic PTV margins [21]. For radiobiological comparison, total prescribed doses by dose level were converted to their equivalent dose in 2 Gy fractions (EQD_2_,Table 1). Online adaptive planning (adapt-to-shape) was performed before each fraction to account for daily anatomical variations. Organs-at-risk (OAR) dose constraints were strictly adhered to, while aiming for a PTV V95% > 99%. No specific patient instructions were given regarding bladder or bowel filling.

**Table 1:** Radiotherapy interventions. Abbreviations: GTV, gross tumor volume; D, total dose; EQD2, equivalent dose in 2 gray fractions.; Gy, Gray.* Not evaluated in this trial.

**Radiotherapy intervention**	**Regime**		**Cumulative GTV dose**	
	Fractions x dose [Gy]		D	EQD_2_
	Week 1	Week 2	[Gy]	[Gy_10_]
Standard treatment	5 x 5		25	31
Dose level 0	5 x 5	2 x 5	35	44
Dose level 1	5 x 5	3 x 5	40	50
Dose level 2	5 x 5	4 x 5	45	56
Dose level 3*	5 x 5	5 x 5	50	63

### Follow-up and toxicity assessment

Toxicity was assessed at baseline, weekly during radiotherapy, and biweekly up to 20 weeks post-treatment or until surgery. Evaluated symptoms included bowel complaints such as proctitis, rectal pain and bleeding, as well as urinary symptoms, fatigue, and dermatitis graded using CTCAE v5.0. In accordance with Dutch colorectal cancer guidelines, response assessments using MRI and endoscopy were performed at weeks 11–13 and, in cases of (near) complete response, repeated at weeks 16–20. Clinical complete response was defined as the absence of residual tumor on MRI, endoscopy, and digital rectal examination, while near-complete response was defined as minimal residual abnormalities considered compatible with ongoing response. Clinical complete and near-complete responders were considered for non-operative management; all others were referred for surgery.

### Dose level assessment

Two types of DLT were defined: (1) acute DLT, which included CTCAE grade 4 toxicity within 20 weeks or grade 3 persisting beyond 12 weeks, or delay of surgery > 20 weeks due to toxicity; (2) perioperative DLT, defined as any Clavien-Dindo grade ≥ 3b complication occurring within 30 days of surgery, provided that surgery was performed within 26 weeks of radiotherapy initiation. A dose level was considered safe if 0 out of 6 patients experienced an acute DLT and fewer than 2 out of 6 experienced a perioperative DLT. If 1 acute DLT and/or 2 perioperative DLTs were observed, the dose level was expanded with 3 additional patients. The dose level was then deemed safe if no further DLTs occurred.

### Statistical analysis

All patients who initiated treatment comprised the analysis set. Baseline patient and tumor characteristics, and follow-up milestones, were presented on a per-patient basis and stratified by dose level. Doses were converted to EQD_2_ (α/β = 10 Gy) using the standard linear-quadratic model. Toxicity and technical metrics were summarized by dose level using descriptive statistics. Continuous variables were reported as median with interquartile range (IQR) or mean with standard deviation (SD), as appropriate; categorical variables as n (%). For toxicity, weekly prevalence for each symptom per CTCAE grade was plotted by dose level. Boost fractions were considered technically acceptable if the PTV V95% was > 90% for both GTV and lymph nodes. Analyses were performed in R (R Foundation for Statistical Computing) using RStudio (2023.03.0–386).

## Results

**Fig. 2:**
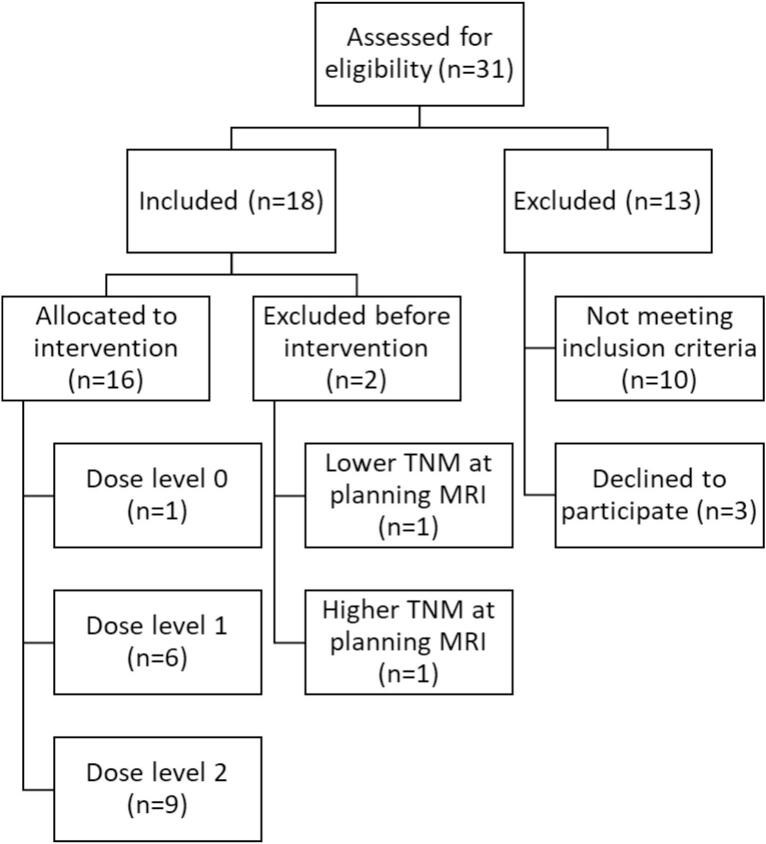
Flow diagram of participant eligibility and recruitment assessment.

Thirty-one patients were referred for SCRT for intermediate-risk rectal cancer during the study period (November 2021 − March 2024). After screening, 21 initially met the eligibility criteria. Three eligible patients declined trial participation. Two patients were excluded after inclusion and before treatment when the planning MRI for radiotherapy revealed ineligibility due to updated disease status. A total of sixteen patients proceeded to trial treatment: one at dose level 0, six at dose level 1, and nine at dose level 2(Fig. 2). At dose level 1, all patients were male and their median age (IQR) was 68 years (58–71). At dose level 2, 33% were male (67% female) and the median age (IQR) was 73 years (65–77 years; Table 2).

**Table 2:** Baseline patient and tumor characteristics divided by dose level enrollment. *Measured at planning MRI. ** Shortest distance between anorectal junction and tumor (planning MRI). Abbreviations: BMI, body mass index; PS, performance status; CCI, Charlson comorbidity index; GTV, gross tumor volume; ARJ, anorectal junction.

**Dose level**	**Patient #**	**Age**	**Sex**	**BMI**	**PS**	**CCI**	**Smoker status**	GTV* (cc)	ARJ – tumor** (cm)	**cTNM**
0	1	56	Male	33	0	2	former	15	6.5	cT2N1a
1	1	69	Male	28	0	4	non	18	3.0	cT3cN1a
	2	55	Male	24	0	3	current	4	6.0	cT2N1b
	3	48	Male	23	0	2	current	38	4.0	cT3bN1a
	4	81	Male	23	0	6	current	12	7.0	cT3aN1a
	5	67	Male	34	0	4	current	7	3.5	cT3aN1b
	6	71	Male	29	0	6	current	12	4.7	cT3bN1a
2	1	73	Male	24	0	5	non	20	1.5	cT3bN1a
	2	80	Male	23	0	6	non	25	2.0	cT3cN0
	3	43	Female	35	0	2	non	43	6.2	cT3bN1b
	4	73	Male	24	0	5	non	12	5.0	cT3aN1a
	5	76	Female	26	1	5	former	11	6.5	cT3bN1a
	6	82	Female	20	0	6	non	13	2.7	cT3bN1a
	7	77	Female	22	1	5	current	11	5.0	cT3bN1a
	8	49	Female	26	0	2	former	30	4.2	cT3dN0
	9	65	Female	28	0	5	current	7	6.3	cT3aN1a

### Dose-limiting toxicity

No acute DLTs were observed at any dose level. At dose level 1, one patient experienced a perioperative DLT. This patient required ICU admission due to perineal wound sepsis following abdominoperineal resection classified as Clavien-Dindo Grade 4a. At dose level 2, two patients experienced perioperative DLTs due to reoperation after low anterior resection (LAR) for anastomotic leakage requiring bowel diversion, both classified as Clavien-Dindo grade 3b. Following cohort expansion and completion of follow-up, DLT incidence at dose level 2 was 2/9, below the stopping threshold.

### Toxicity

**Fig. 3:**
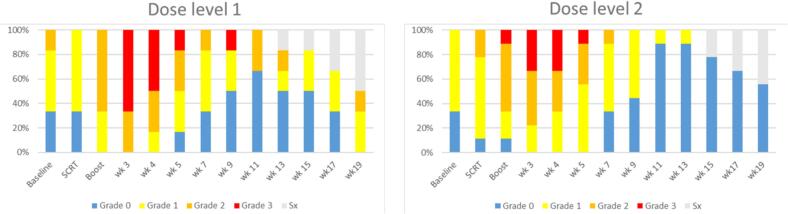
The overall highest grade of toxicity by weekly interval in both dose level 1 (left column, n = 6) and 2 (right column, n = 9). On the x-axis is the follow-up moments indicated, and on the y-axis the symptom grade percentage. The grey bars are patients censored after surgery (Sx). There was no lost-to-follow-up.

Proctitis was the most frequently reported toxicity and accounted for most instances of the overall highest grade of toxicity (Fig. 3), followed by fatigue. Proctitis was the only toxicity reaching grade 3; no grade ≥ 3 events occurred for any other toxicity. Across dose levels, toxicity prevalence peaked in weeks 2–4 after radiotherapy initiation and declined by weeks 5–7 (Fig. 3; Suppl. Fig. 1). The single patient at dose level 0 experienced grade 1 proctitis during the two treatment weeks and grade 3 in week 3, accompanied by grade 1 fatigue, rectal bleeding, and cystitis. At dose levels 1 and 2, grade 3 proctitis occurred in 4/6 (67%) and 5/9 (56%) patients, respectively. All proctitis grade 3 events (n = 11) were self-limited without medical intervention or hospitalization: four events (36%) resolved within 1–7 days, and seven (64%) within 8–14 days. Additional toxicities at dose level 1 included grade 1 abdominal pain (n = 4), grade 1 spermatic cord hemorrhage (n = 1), grade 2 anorexia (n = 1), and grade 2 constipation (n = 1), whereas no additional toxicities were reported at dose level 2.

### Tumor response

The single patient at dose level 0 (n = 1) had TME surgery after a partial response at the first response evaluation. At dose level 1 (n = 6), three patients had TME surgery due to partial response at the first evaluation, two patients had TME surgery due to partial response at the second evaluation, and one patient was still under surveillance at 26 weeks. At dose level 2 (n = 9), four patients underwent TME surgery due to partial response at first evaluation, while five patients remained under surveillance at 26 weeks. Among operated patients, procedures included six low anterior resections (LAR) and three abdominoperineal resections (APR). All four LAR procedures at dose level 1 were performed with a diverting stoma, whereas the two LAR procedures at dose level 2 were performed without diversion. Among the patients who underwent TME, two had achieved a pathological complete response at dose level 2. The organ preservation rate of dose level 0 to 2 at 26 weeks were 0%, 17%, and 56% respectively. Overall, the median interval (interquartile range) from start of radiotherapy to surgery was 17 weeks (IQR: 15–20 weeks). The patient outcomes per dose level are summarized in Table 3.

**Table 3:** Outcome of each patient per dose level with their status at 26 weeks, operations when performed within 26 weeks after start radiotherapy, and DLT incidence. Abbreviations: DLT, dose-limiting toxicity.

**Dose level**	**Patient #**	**Baseline cTNM**	**Patient status at 26 weeks**	**Acute toxicity DLT**	**Surgery**		**Perioperative DLT**
					**Week**	**ypTNM**	
0	1	cT2N1a	Operated	No	20	ypT3N2a	No
1	1	cT3cN1a	Operated	No	22	ypT2N2a	No
	2	cT2N1b	Operated	No	18	ypT1N0	No
	3	cT3bN1a	Operated	No	12	ypT2N0	No
	4	cT3aN1a	Operated	No	23	ypT2N1c	Yes
	5	cT3aN1b	Operated	No	16	ypT1N0	No
	6	cT3bN1a	Surveillance	No			
2	1	cT3bN1a	Surveillance	No			
	2	cT3cN0	Operated	No	17	ypT0N0	No
	3	cT3bN1b	Operated	No	14	ypT2N0	Yes
	4	cT3aN1a	Operated	No	14	ypT0N0	Yes
	5	cT3bN1a	Surveillance	No			
	6	cT3bN1a	Surveillance	No			
	7	cT3bN1a	Operated	No	19	ypT2N0	No
	8	cT3dN0	Surveillance	No			
	9	cT3aN1a	Surveillance	No			

### Technical feasibility

All boost fractions were technically acceptable for both GTV and involved lymph nodes. At dose level 0, V95% coverage for tumor and nodal PTVs were 99.8% and 98.8%, respectively. For dose level 1, median tumor PTV V95% was 99.0% (IQR: 98.7–99.4%), and 98.6% (IQR: 97.5–99.1%) for pathological lymph nodes. At dose level 2, median tumor PTV V95% was 99.1% (IQR: 97.3–99.4%), and 95.4% (IQR: 86.0–99.1%) for nodal volumes. Organ-at-risk (OAR) constraints were prioritized and consistently met across all treatments.

## Discussion

This phase I trial evaluated the safety and feasibility of dose-escalated, online adaptive MR-guided SCRT for patients with intermediate-risk rectal cancer. The MTD was not identified. Dose level 2, SCRT followed by four additional 5 Gy boost fractions, was the highest dose level completed without exceeding prespecified DLT stopping rules and OAR constraints. Technical feasibility was acceptable for all adaptive boost fractions. Accordingly, dose level 2 was considered sufficiently safe and technically feasible for further evaluation.

Reported rates of acute grade ≥ 3 toxicity after rectal radiotherapy vary widely across regimens and with concurrent chemotherapy use. In the current trial, all radiotherapy-related grade 3 events were self-limited proctitis peaking in weeks 3–4, and no acute DLTs occurred. Consistent with prospective SCRT-delay data, temporary moderate bowel symptoms commonly peak during the early post-SCRT interval, whereas severe or life-threatening events are uncommon, supporting overall tolerability [27]. In a large prospective high-field MR-linac cohort, acute grade ≥ 3 toxicity was rare after online adaptive MR-guided SCRT [25]. The comparatively higher incidence of grade 3 proctitis observed here likely reflects the intended boost-driven increase in rectal mucosal dose, yet remained reversible and within the acute DLT stopping boundaries.

Perioperative safety was evaluated using Clavien-Dindo ≥ 3b complications. Overall, 3 events (1 in dose level 1, and 2 in dose level 2) were observed among 10 operated patients. In randomized controlled trials combining SCRT with neoadjuvant systemic therapy, such as STELLAR and RAPIDO, 30-day Clavien-Dindo grade ≥ 3 complication rates were 14% and 18%, respectively [28], [29]. Although Stockholm III (SCRT) did not report Clavien-Dindo grades, delaying surgery by 4–8 weeks reduced overall 30-day postoperative complications (41% vs 53% with immediate surgery) without a significant increase in anastomotic leakage (9%−11%) [16]. Notably, in the current trial, three patients underwent LAR without a diverting colostomy, and two required reoperation for anastomotic leakage. The number of dose level 2 patients undergoing surgery within 26 weeks was smaller than anticipated, yielding a high event proportion in this small subgroup. While numbers are small, these findings raise the question of whether a temporary diverting colostomy should be routinely considered following a TME-LAR after radiotherapy dose escalation and warrant prospective evaluation.

Non–MR-guided dose-escalation strategies in rectal cancer have shown mixed effects on complete response and organ-preservation outcomes. In the randomized RECTAL-BOOST trial, an external-beam boost added to standard chemoradiotherapy did not improve complete response rates in locally advanced rectal cancer [18]. In contrast, the OPERA trial demonstrated that a highly focal contact X-ray brachytherapy boost could improve long-term organ preservation in selected early rectal cancer patients, albeit at the expense of increased local mucosal toxicity [19].

Other organ-preservation approaches, particularly total neoadjuvant therapy (TNT), primarily intensify systemic treatment and have improved response rates and systemic control. In the OPRA trial, structured response assessment after TNT resulted in a 3-year organ-preservation rate of 53%, while phase III TNT trials such as RAPIDO and PRODIGE-23 demonstrated improved systemic control and increased pathological complete response rates compared with conventional chemoradiotherapy-based strategies [13], [28], [30].

The current trial evaluated a different strategy focused on local radiotherapy intensification using online adaptive MR-guided radiotherapy after SCRT, aiming to escalate tumor dose while maintaining organ-at-risk constraints. In the highest completed dose level, the 26-week organ-preservation rate was 56% (5/9). Although follow-up remains limited and patient populations differ substantially from other organ-preservation studies, these findings support further investigation of MR-guided dose escalation strategies in larger prospective trials. The ongoing STARTREC 3 phase II trial (CTIS 2024–514620-17–00), which includes a four-boost-fraction strategy similar to the current trial, is now underway to further investigate treatment response in a broader population of early- and intermediate-risk rectal cancer [31].

A limitation was premature trial closure due to changes in national TNM staging guidelines, which reduced accrual and precluded escalation beyond dose level 2. In addition, response assessment was not centralized in this trial, which could have resulted in surgery in patients with a ypCR. These findings also underline the challenges of multimodal response assessment after radiotherapy dose escalation and emphasize the importance of centralized response assessment by an experienced multidisciplinary team. Despite these limitations, escalation through dose level 2 met prespecified safety stopping rules and 26-week organ preservation rates increased across the dose levels; an exploratory finding compatible with a dose–response effect.

In conclusion, online adaptive MR-guided SCRT followed by a GTV boost of 4 × 5 Gy is safe and technically acceptable for intermediate-risk rectal cancer and merits further evaluation as an organ-preservation strategy.

## Declaration of generative AI and AI-assisted technologies in the manuscript preparation process

During the preparation of this work the author(s) used ChatGPT (OpenAI, San Francisco, CA, USA) in order to improve grammar. After using this tool/service, the author(s) reviewed and edited the content as needed and take(s) full responsibility for the content of the published article.

## CRediT authorship contribution statement

**H. Eijkelenkamp:** Writing – review & editing, Writing – original draft, Visualization, Resources, Methodology, Investigation, Formal analysis, Data curation, Conceptualization. **M.E. Verweij:** Writing – review & editing, Project administration, Methodology, Investigation, Data curation, Conceptualization. **G. Grimbergen:** . **M. Tanaka:** Writing – review & editing, Methodology, Data curation, Conceptualization. **C.M. Kensen:** Writing – review & editing, Conceptualization. **T. Janssen:** Writing – review & editing, Conceptualization. **T. Vijlbrief:** Writing – review & editing, Conceptualization. **W.M.U. van Grevenstein:** Writing – review & editing, Conceptualization. **L.M.G. Moons:** Writing – review & editing, Conceptualization. **M. Koopman:** Writing – review & editing, Conceptualization. **M.M. Lacle:** Writing – review & editing, Conceptualization. **M.N.G.J.A. Braat:** . **M. Maas:** Writing – review & editing, Conceptualization. **E.C.J. Consten:** . **A. Pronk:** Writing – review & editing, Conceptualization. **A.B. Smits:** Writing – review & editing, Conceptualization. **J.T. Heikens:** Writing – review & editing, Conceptualization. **S.G. Elias:** Writing – review & editing, Conceptualization. **H.M. Verkooijen:** Writing – review & editing, Conceptualization. **G.J. Meijer:** Writing – review & editing, Conceptualization. **A. Couwenberg:** Writing – review & editing, Conceptualization. **M.P.W. Intven:** Writing – review & editing, Supervision, Resources, Project administration, Methodology, Investigation, Conceptualization.

## Funding

This research received financial support for monitoring costs through the MOMENTUM study (NCT04075305). No specific grant was received from funding agencies in the public, commercial, or not-for-profit sectors.

## Declaration of Competing Interest

The authors declare that they have no known competing financial interests or personal relationships that could have appeared to influence the work reported in this paper.

## Appendix A Supplementary data

Supplementary Data 1 Toxicity-specific weekly prevalence by grade (dose levels 1–2).
