# Supplementary material for: Safety and early organ preservation outcomes after dose escalation with MR-guided radiotherapy for rectal cancer (preRADAR): A phase I trial
Source: Clin Transl Radiat Oncol. 2026 May 25;60:101191. doi: 10.1016/j.ctro.2026.101191 (PMC13263622; doi:10.1016/j.ctro.2026.101191)
Supplement: Supplementary Data 1 — Toxicity-specific weekly prevalence by grade (dose levels 1–2). [file mmc1.docx]

## Supplementary


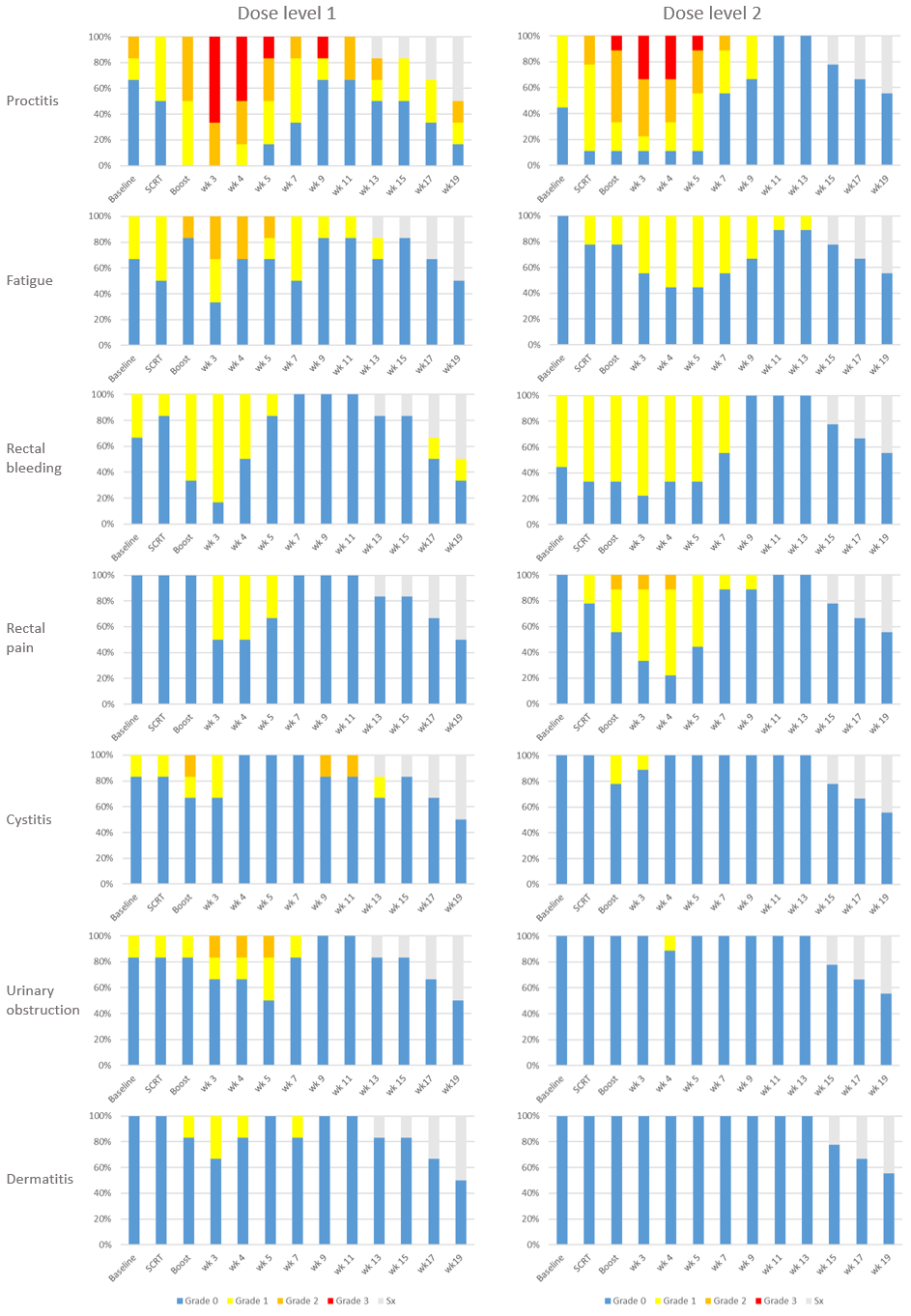


Figure 3. The seven most reported toxicities in both dose level 1 (left column, n=6) and 2 (right column, n=9). On the x-axis is the follow-up moments indicated, and on the y-axis the symptom grade percentage. The grey bars are patients censored after surgery (Sx). There was no lost-to-follow-up.
